# Giant desmoplastic small round cell tumor of the abdomen: A case report

**DOI:** 10.3389/fonc.2022.992346

**Published:** 2022-11-22

**Authors:** Wuke Wang, Yunjie Chen, Chunnian Wang, Hui Su

**Affiliations:** ^1^ Department of General Surgery, Hwa Mei Hospital, University of Chinese Academy of Sciences, Ningbo No. 2 Hospital, Ningbo, China; ^2^ Department of Gastrointestinal Pathology, Ningbo Diagnostic Pathology Center, Ningbo, China; ^3^ Department of Gastrointestinal Surgery, First Affiliated Hospital of Jinan University, Guangzhou, China

**Keywords:** desmoplastic small round cell tumor, abdominopelvic tumor, medium-elderly male, DSRCT, case report

## Abstract

**Background:**

Desmoplastic small round cell tumor (DSRCT) is a rare, aggressive, mesenchymal malignancy of a separate clinicopathological entity. It has a predilection for young men, with no evidence of any ethnic predilection. The current diagnostic gold standard for DSRCT includes histopathologic, immunohistochemical, and cytogenetic studies in order to confirm the variable phenotypic expression and characteristic chromosomal translocation.

**Case summary:**

A 65-year-old man presented with a sensation of an abdominal mass and a presentation of an incomplete bowel obstruction. Initial lab tests were in the normal range except for carbohydrate antigen. Contrast-enhanced CT showed that a large, mass-confounding density was occupied in the omentum majus area of the middle and lower abdominal wall. A 3D reconstruction of the images was performed to clarify the relationship between the tumor and the colon and was confirmed by a colonoscopy. After surgery, immunohistochemistry and fluorescence *in situ* hybridization (FISH) revealed EWSR1-WT1 gene rearrangement at 22q12, confirming the diagnosis of desmoplastic small round cell tumor.

**Conclusion:**

Being different from the predilection of DSRCT for young men, the patient in our case is a 65-year-old man with a huge mass involving the transverse colon and the bladder.

## Introduction

The desmoplastic small round cell tumor (DSRCT) is a rare, aggressive, mesenchymal malignancy of uncertain differentiation with both a characteristic chromosomal translocation and immunohistochemical profile (1). Initially described in 1989 by Gerard and Rosai, DSRCT tumor cells were classified by express epithelial, neuronal, and skeletal muscle and mesenchymal markers. However, by 1991, DSRCT was formally established as a separate clinicopathological entity (2). It has a predilection for young men, with ages ranging from 3 to 52 years, and with a peak incidence between the second and third decades of life. The mean age at diagnosis is 20.8 years, and it has a male-to-female ratio of 10:1, with no evidence of any ethnic predilection. The most common symptom of DSRCT is vague abdominal symptoms (3), and the more common is terminal disease on diagnosis with multiple intra-abdominal lesions (4). The current diagnostic gold standard for DSRCT includes histopathologic, immunohistochemical, and cytogenetic studies in order to confirm variable phenotypic expression and characteristic chromosomal translocation (5).

## Case presentation

A 65-year-old man presented with a sensation of an abdominal mass and a presentation of an incomplete bowel obstruction. He had a previous history of hypertension and hyperlipidemia; percutaneous internal coronary stenting was performed 9 years ago, and he is currently taking betaloc 47.5 mg, aspirin 100 mg, and rosuvastatin 10 mg, without a remarkable family history. Physical examination showed that his vital signs were within normal range. An abdominal examination could reach a massive mass extending from the middle abdomen to the pelvic cavity, which is about 16 cm in size, poor in mobility, and tough in texture; the rest abdomen was soft and non-tender, without signs of peritoneal irritation. Initial lab tests including complete blood count and tumor markers presented in the **Table 1**.

**Table 1:** Leucocyte count was 12.9*10^9/L; neutrophil ratio was 86.9%, higher than the normal values; carbohydrate antigen was 125 216.30 U/ml, significantly higher than the normal values.

Inspection item	Result	Reference ranges	Unit
Leucocyte count	12.9	3.5–9.5	*10^9/L
Classification of neutrophils	86.9	40.0–75.0	%
Absolute values of the monocytes	0.62	0.1–0.6	*10^9/L
Eosinophil count	0.01	0.02–0.52	*10^9/L
RBC	4.55	4.30–5.80	*10^12/L
Hemoglobin	134	130–175	g/L
Platelet count	233	125–350	*10^9/L
AFP	3.5	≤7.00	ng/ml
Carcinoembryonic antigen	1.04	≤5.00	ng/ml
Ferritin	164.1	22.0–322.0	ng/ml
Carbohydrate antigen 125	216.3	≤15.00	U/ml
Carbohydrate antigen 19-9	12.55	≤34.00	U/ml
Composite prostate antigen	0.78	<3.600	ng/ml
Free prostate-specific antigen	0.51	<0.930	ng/ml
Total prostate-specific antigen	1.29	<4.000	ng/ml
Carbohydrate antigen 50	9.08	<25.00	U/ml
Carbohydrate antigen 242	13.27	<25.00	U/ml
Carbohydrate antigen 72-4	1.6	<10.00	U/ml
Neuron-specific enolase	9.99	<20.00	ng/ml
Cytokeratin 19 fragment	1.86	<3.30	ng/ml
Squamous cell carcinoma antigen	0.26	<1.50	ng/ml
Acquisition time:	2022/4/25 15:06	Check time:	2022/4/26 13:58

Other tumor indicators were in the normal range. Red color value means higher than normal value, and blue color value means lower than normal value.

**Figure 1:**
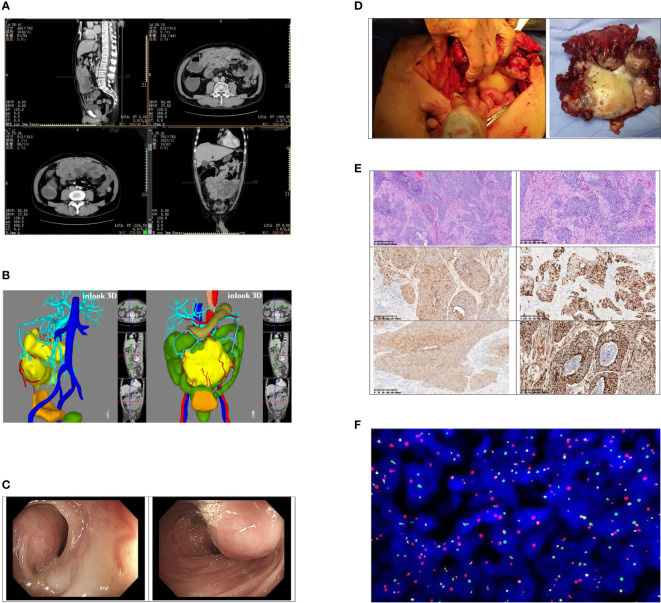
**(A)** Contrast-enhanced CT found that the larger cross-section range is about 167*149*65 mm; moderate uneven enhancement occurred after the enhancement; obscure boundary, unresolved from the adjacent transverse colon and part of the small intestine. Multiple effusion dilations were seen in the ascending colon and part of the small intestine. **(B)** The position of the tumor in the abdominal cavity and its relationship with various organs were displayed by 3D reconstruction imaging of CT. **(C)** A huge external pressure mass under the mucosa at the middle section of the transverse colon (approximately 65 cm away from the anus) made the intestinal lumen compressed, twisted, and narrow. **(D)** The tumor involved the middle part of the transverse colon and part of the bladder wall. The size was about 18 * 12 * 6 cm. **(E)** The pictures of tumor pathology sections; order is: ×40, ×100, CKpan (+), desmin (+), NSE (+), vim (+). **(F)** Project name: EWSR1/WT1 fusion gene test (FISH-tissue). Detection probe: LBPEWSR1/WT1 fusion gene probe. Chromosome loci: WTl (llpl3)/EWSRl (22ql2). Probe tag: green signal is GSPEWSR1, red signal (R) is the GSPWT1 detection result. Cell number analyzed: 200; Diagnostic opinion: The EWSR1/WT1 fusion gene was detected, 200 interval phase cells were analyzed, and each signal pattern was as follows: 1G1R1F39.0%, 1G1R2F20.0%, 1G1F4.0%, lG2F 5.0%, lRlF 5.0%, lR2F 4.0%, lGlR 9.0%, 2GlR 5.0%, lG2R 4.0%, 2G2R 5.0%.

Contrast-enhanced CT of the abdomen showed that a large, mass-confounding density was occupied in the omentum majus area of the middle and lower abdominal wall (**Figure 1A**). A 3D reconstruction of the images was performed (**Figure 1B**). To clarify the relationship between tumor and colon, colonoscopy was performed (**Figure 1C**).

The preoperative diagnosis of the patient was intestinal obstruction and abdominal tumor, and he underwent surgery in May this year. During the operation, we observed that the tumor originated from the omentum majus and invaded the middle segment of the transverse colon, the anterior wall of bladder, and the abdominal wall. No other distant metastasis was found. The patient underwent surgical treatment that included a 5-cm transverse colon at each end with the mass involvement, the involved bladder tissue, and the lower umbilical range of about 10 * 10 cm of the adhesion peritoneum and rectus abdominal sheath. The complete tumor was removed and elevated colostomy was performed. (**Figure 1D**) The operation time of the patient was 282 minutes, the intraoperative bleeding was 100 ml, and there was no postoperative complication. Postoperatively, the patient was generally in a stable condition and discharged 9 days later.

The size of the tumor was 18 * 12 * 6 cm. The cut surface of the tumor was gray, with hemorrhage in the center. Histologically, the neoplasm was lobulated and consisted of small round cells with amphiphilic cytoplasm and round to ovoid mononuclear hyperchromatic nuclei. There was marked mitosis and necrosis. The neoplasm involved the surrounding adipose tissue and was infiltrated into the intestinal and bladder wall. (**Figure 1E**) Immunohistochemistry showed that the tumor cells differentiated into epithelium, muscle, and nerve. Tumor cells expressed an epithelial marker, such as CK(pan), CAM5.2, and EMA. Desmin, NSE, vimentin, and CD99 proteins were strongly expressed in the tumor cells. The positive rate of Ki-67 was about 60%. Some tumor cells expressed MC(HBME1). However, GATA-3, P63, CK5/6, Syn, CgA, CEA, Wilms tumor, MyoD1, myogenin, NKX2.2, calretinin, CD56, S-100, SOX-10, and PGP9.5 were negative. Fluorescence *in situ* hybridization (FISH) revealed EWSR1-WT1 gene rearrangement at 22q12, confirming the diagnosis of a desmoplastic small round cell tumor (**Figure 1F**). In August, the patient was reexamined with abdominal enhanced CT and chest CT, and was found with no tumor recurrence and metastasis. The patient has received chemotherapy four times since the operation. The chemotherapy regimen was oral dacarbazine 0.5 g d1–d4 and intravenous doxorubicin hydrochloride liposome 50 mg d1.

## Discussion

The desmoplastic small round cell tumor (DSRCT) is a soft tissue malignant neoplasm of the small round cell tumor family which occurs mainly in the abdominal and pelvic cavity of young patients (1). DSRCT has unique histology and immunohistochemical and molecular biology features, and is characterized by abdominopelvic sarcoma (6), including multi-lineage cellular nests of mesenchymal, epithelial, muscular, and neural differentiation admixed with desmoplastic stroma (7). In typical cases, the tumor is composed of oval cells with high nuclear to cytoplasmic ratio, which can also show striated features or clear cytoplasm, resulting in signet ring cells in a few cases. The exact incidence rate of DSRCT is unclear, although at least 1000 cases have been reported in the literature since its histopathological description. The 5-year overall survival rate in the retrospective study was approximately 10% (1, 8). Clinically, the signs and symptoms of DSRCT are no specific symptoms and most patients present with a single abdominal mass, as in our case. It is sometimes associated with pain, abdominal distention and/or ascites, constipation, weight loss, or other symptoms secondary to an extrinsic mass effect (bowel obstruction) or due to a compromise of abdominopelvic organs. This cancer is believed to originate from the surface of the peritoneum and to metastasize almost universally when it occurs. The common sites of metastasis include the liver, spleen, and lymph nodes above the diaphragm (9), although the disease may occur in different sites, including the testis and the central nervous system (10). DSRCT has many different staging methods, and the most recent uses imaging characteristics to define intermediate (no liver involvement or ascitic fluid), high-risk (either liver involvement or ascitic fluid), and very-high-risk disease (both liver involvement and ascitic fluid) (3).

Immunohistochemically, DSRCT had an immune spectrum of polyphenols with tumor cells expressing epithelium, mesenchymal, and neuroendocrine markers (11). Due to DSCRT and other tumor types, the final diagnosis depends on cytogenetics and molecular analysis of *in situ* hybridization or reverse transcription polymerase chain reaction. The specific molecular feature of DSRCT is the pathognomonic EWSR1-WT1 t(11;22) (p13:q12) translocation (12). In addition to the translocation defined by this disease, the understanding of recurrent carcinogenic changes or DSRCT subgroups defined by the genome is still limited (13). The genome-wide sequencing of DSRCT samples did not show any other information about secondary driving carcinogenic events other than the ewsr1–wt1 fusion. WT1 is considered as a useful antibody to diagnose DSRCT and distinguish it from other tumors with small blue cell morphology. Therefore, molecular detection is recommended to avoid diagnostic traps. We performed FISH analysis on this case to determine whether there was an ewsr1 gene break. Not surprisingly, the ewsr1 division signal was detected in tumor cells, which finally confirmed the diagnosis of DSRCT.

DSRCT is notorious for extensive metastasis. Patients usually have obvious tumor burden at the initial examination. The symptoms are not obvious until the peritoneal surface is widely infiltrated by the tumor, and no definite operation can be performed (14). Given the rarity of this disease, no randomized trials addressing its treatment have been performed, and nearly all the available literature describes anecdotal or retrospective experiences. Therefore, there is no standard treatment at present. The treatment methods that have been used include high-dose alkylating agent chemotherapy and trial complete cytoreductive surgery. Other consolidation local control methods include the use of radiolabeled antibodies for research treatment and hyperthermic intraperitoneal chemotherapy (HIPEC). Despite intensive, multimodal treatment, recurrence is still common. Although, so far, the treatment methods for potential fusion oncogenes in DSRCT are not clear, drug development for this and other similar central drivers is still possible (15).

## Data availability statement

The original contributions presented in the study are included in the article/supplementary material. Further inquiries can be directed to the corresponding author.

## Author contributions

All persons who meet authorship criteria are listed as authors, and all authors certify that they have participated sufficiently in the work to take public responsibility for the content, including participation in the concept, design, analysis, writing, or revision of the manuscript.

## Funding

Funded by the Project of NINGBO Leading Medical & Health Discipline, Project Number: 2022-F19. Supported by Ningbo Natural Science Foundation, China (Grant No.2019A610215).

## Conflict of interest

The authors declare that the research was conducted in the absence of any commercial or financial relationships that could be construed as a potential conflict of interest.

## Publisher’s note

All claims expressed in this article are solely those of the authors and do not necessarily represent those of their affiliated organizations, or those of the publisher, the editors and the reviewers. Any product that may be evaluated in this article, or claim that may be made by its manufacturer, is not guaranteed or endorsed by the publisher.

## References

[1] Sanguino A Kaur G Mao S . Desmoplastic small round-cell tumor: Retrospective review of institutional data and literature review. Anticancer Res (2021) 41(8):3859–66. doi: 10.21873/anticanres.15179 34281846

[2] de Alava E Marcilla D . Birth and evolution of the desmoplastic small round-cell tumor. Semin Diagn Pathol (2016) 33(5):254–61. doi: 10.1053/j.semdp.2016.05.003 27312937

[3] Saltsman JA 3rd Price AP Goldman DA Hammond WJ Danzer E Magnan H . A novel image-based system for risk stratification in patients with desmoplastic small round cell tumor. J Pediatr Surg (2020) 55(3):376–80. doi: 10.1016/j.jpedsurg.2018.02.068 PMC6126997 29605262

[4] Slim S Zemni I Bouida A Bouhani M Boujelbene N Mrad K . Intraabdominal and ganglionic desmoplastic small round cell tumor: A case series. J Med Case Rep (2021) 15(1):500. doi: 10.1186/s13256-021-03094-9 34635162 PMC8507229

[5] Wei G Shu X Zhou Y Liu X Chen X Qiu M . Intra-abdominal desmoplastic small round cell tumor: Current treatment options and perspectives. Front Oncol (2021) 11::705760. doi: 10.3389/fonc.2021.705760 34604040 PMC8479161

[6] Ertoy Baydar D Armutlu A Aydin O Dagdemir A Yakupoglu YK . Desmoplastic small round cell tumor of the kidney: A case report. Diagn Pathol (2020) 15(1):95. doi: 10.1186/s13000-020-01015-w 32703229 PMC7379352

[7] Thway K Noujaim J Zaidi S Miah AB Benson C Messiou C . Desmoplastic small round cell tumor: Pathology, genetics, and potential therapeutic strategies. Int J Surg Pathol (2016) 24(8):672–84. doi: 10.1177/1066896916668637 27621277

[8] Loktev A Shipley JM . Desmoplastic Small round cell tumor (DSRCT): Emerging therapeutic targets and future directions for potential therapies. Expert Opin Ther Targets (2020) 24(4):281–5. doi: 10.1080/14728222.2020.1738392 32125905

[9] Jin D Chen M Wang B Gou Y . Mediastinal desmoplastic small round cell tumor. Med (Baltimore) (2020) 99(44):e22921. doi: 10.1097/MD.0000000000022921 PMC7598786 33126354

[10] Sedig L Geiger J Mody R Jasty-Rao R . Paratesticular desmoplastic small round cell tumors: A case report and review of the literature. Pediatr Blood Cancer (2017) 64(12). doi: 10.1002/pbc.26631 28509382

[11] Magro G Broggi G Zin A Di Benedetto V Meli M Di Cataldo A . Desmoplastic small round cell tumor with "Pure" spindle cell morphology and novel EWS-WT1 fusion transcript: Expanding the morphological and molecular spectrum of this rare entity. Diagnostics (Basel) (2021) 11(3):545. doi: 10.3390/diagnostics11030545 33803887 PMC8003219

[12] Gundem G Gerstle JT Heaton TE LaQuaglia MP Wexler LH Meyers PA . Comprehensive molecular profiling of desmoplastic small round cell tumor. Mol Cancer Res (2021) 19(7):1146–55. doi: 10.1158/1541-7786.MCR-20-0722 PMC8293793 33753552

[13] Dundr P Drozenová J Matěj R Bártů M Němejcová K Robová H . Desmoplastic small round cell tumor of the uterus: A report of molecularly confirmed case with EWSR1-WT1 fusion. Diagnostics (Basel) (2022) 12(5):1184. doi: 10.3390/diagnostics12051184 35626339 PMC9140206

[14] Hayes-Jordan A LaQuaglia MP Modak S . Management of desmoplastic small round cell tumor. Semin Pediatr Surg (2016) 25(5):299–304. doi: 10.1053/j.sempedsurg.2016.09.005 27955733 PMC5614508

[15] Silva MLG Torrezan GT Costa FD Formiga MN Nicolau U Nascimento AG . Desmoplastic small round cell tumor: A review of main molecular abnormalities and emerging therapy. Cancers (Basel) (2021) 13(3):498. doi: 10.3390/cancers13030498 33525546 PMC7865637

